# Surgery of the Ancients

**Published:** 1816-12

**Authors:** 


*For the London Medical and Physical Journal.*

### *Surgery of the Ancients.*

**[From our Parisian Correspondent.]**

MANY contradictory opinions have been omitted as to the knowledge of the ancients in the art of Surgery. The progress of the French arms in Egypt tend to throw some light upon the subject. After the obstinate battle of Sedment, says Baron Larrey, General Desaix pursued the enemy beyond the Cataracts, and afforded the Commission of the Arts the facility of visiting the monuments of ancient Thebes, with her hundred gates. The renowned temples of Tentyra, of Carnate, and Luxor, preserve, even in their ruins, splendid proofs of her ancient magnificence. On the ceilings and walls of these temples are found bas-reliefs, representing members cut with instruments similar to those used by modern surgery in amputations. The same instruments are found in hieroglyphics; and we discovered the traces of other surgical operations, which *prove* that surgery, in these remote ages, marched in a line with the other arts, whose perfection appears to have been carried to a very high degree.

